# 3D-Geomorphometrics tooth shape analysis in hypodontia

**DOI:** 10.3389/fphys.2014.00154

**Published:** 2014-04-23

**Authors:** Ibrahim Al-Shahrani, Wendy Dirks, Nicholas Jepson, Khaled Khalaf

**Affiliations:** ^1^Division of Orthodontics, Department of Preventive Dental Sciences, College of Dentistry, King Khalid UniversityAbha, Saudi Arabia; ^2^Department of Orthodontics, Centre for Oral Health Research, School of Dental Sciences, Newcastle UniversityNewcastle upon Tyne, UK; ^3^Dental School and Hospital, The University of AberdeenAberdeen, UK

**Keywords:** hypodontia, Procrustes analysis, shape analysis, teeth

## Abstract

Assessment of tooth morphology is an important part of the diagnosis and management of hypodontia patients. Several techniques have been used to analyze tooth form in hypodontia patients and these have shown smaller tooth dimensions and anomalous tooth shapes in patients with hypodontia when compared with controls. However, previous studies have mainly used 2D images and provided limited information. In the present study, 3D surface-imaging and statistical shape analysis were used to evaluate tooth form differences between hypodontia and control patients. Eighteen anatomical landmarks were recorded on the clinical crown of the lower left first permanent molar of 3D scanned study models of hypodontia and control subjects. The study sample group comprised of 120 hypodontia patients (40 mild, 40 moderate, and 40 severe hypodontia patients) and 40 age- and sex-matched controls. Procrustes coordinates were utilized to scale and superimpose the landmark coordinate data and then were subjected to principal component analysis (PCA). Subsequently, differences in shape as well as size were tested statistically using allometric analysis and MANOVA. Significant interaction was found between the two factor variables “group” and “sex” (*p* < 0.002). Overall expected accuracies were 66 and 56% for females and males, respectively, in the cross-validated discriminant-analysis using the first 20 PCs. Hypodontia groups showed significant shape differences compared with the control subjects (*p* < 0.0001). Significant differences in tooth crown shape were also found between sexes (*p* < 0.0001) within groups. Furthermore, the degree of variation in tooth form was proportional to the degree of the severity of the hypodontia. Thus, quantitative measurement of tooth shape in hypodontia patients may enhance the multidisciplinary management of those patients.

## Introduction

The term “hypodontia” refers to the congenital absence of one or more teeth. It is the most frequently occurring dental anomaly (Brook, 1984; Dhanrajani, 2002; McKeown et al., 2002; Kirzioglu et al., 2005; Wu et al., 2007). The prevalence of hypodontia in the permanent dentition, excluding third molars, has been estimated to be between 2 and 10% of the population (Polder et al., 2004). The precise etiology and pathogenesis of the congenital absence of teeth is still unclear. However, it appears that it is the result of environmental, epigenetic or genetic factors or a combination of these. Brook (1984, 2009) suggested a multifactorial model in which polygenic factors play a major part but environmental factors are also included. Variations in the size and shape of the remaining teeth have also been found to be associated with hypodontia (Brook, 1984; Schalk-van der Weide et al., 1992; Schalk-van der Weide and Bosman, 1996; McKeown et al., 2002). These dental anomalies can result in various features of malocclusion including spaced dental arches, differences in maxillary and mandibular dental arch lengths, increased overjet and overbite. These features, in addition to the anchorage requirement typically encountered in hypodontia patients, can complicate treatment planning. Accurate knowledge of the size and the exact shape of each tooth using accurate 3D imaging techniques in hypodontia cases will help in reshaping teeth or in determining how much space is needed to be opened to allow the restorative replacement of the missing teeth, in order to achieve harmony in intra- and inter-arch relationships. This will also add valuable information regarding the need to develop a new bracket prescription specifically designed for hypodontia patients. The aim of the current study is to apply a novel 3D geometric morphometric technique to quantify the crown size, shape and allometric variation of lower left first permanent molars of scanned images of study models from hypodontia patients and age and sex matched controls.

Several techniques have been proposed to quantify tooth size and shape. Some of these involve the use of traditional morphometrics such as linear measurements (mesio-distal MD and bucco-lingual BL dimensions) and have revealed smaller tooth dimensions in patients with hypodontia than in controls (Rune and Sarnas, 1974; Schalk-van der Weide et al., 1992; Schalk-van der Weide and Bosman, 1996). The drawbacks of using only linear measurements are that they give limited information, pertinent mainly to tooth size, and do not describe variations in tooth shape or form. Recently, there has been an increased interest in the use of geometric morphometrics (GMM) to study the form of teeth. GMM is a set of statistical tools designed principally for the analysis of biological shape based on landmark coordinates. Shape information is extracted by removing any translational or rotational differences and then scaling to a best fit. Several authors emphasize the ability of geometric morphometric techniques to assess morphological differences precisely and have recommended the use of three-dimensional (3D) tools, avoiding possible complications derived from the analysis of 2D images (Gómez-Robles et al., 2007, 2008).

GMM analyses the relative positions of anatomical landmarks used to approximate the outlines and surfaces of the tested object. The geometric information about shape variation is retained and statistical power is increased. The use of geometric morphometric techniques in conjunction with multivariate analysis has had a great impact on biological studies, since it allows a comprehensive analysis of variations in biological shape. An important feature of these techniques is that they allow the non-destructive 3D capture of the geometry of the morphological structure and preserve this information throughout the analysis (Adams et al., 2004). In addition, geometric methods permit the quantifying of differences in size and in shape which cannot be accomplished using traditional methods (Monteiro et al., 2002). An investigation using 3D GMM of a large number of subjects divided into subgroups of patients with differing degrees of severity of hypodontia analysis is therefore expected to be more reliable and efficient compared to 2D imaging techniques for accurately evaluating tooth morphology in patients with this common dental anomaly and can positively influence our treatment planning, particularly orthodontic treatment, for these patients.

## Materials and methods

### Data collection

This is a retrospective cross-sectional case-controlled study designed to compare the lower first molar crown morphology of the permanent dentition of hypodontia and control subjects using modern three-dimensional geometric morphometric analysis techniques. The study population comprised one hundred and sixty subjects between 12 and 18 years old: a study group consisting of individuals with hypodontia (hypodontia group) and the other with a set of healthy permanent teeth (control group). Subjects were selected from the Hypodontia Patient Database associated with the multi-disciplinary Hypodontia Clinic at Newcastle Dental Hospital. Control patients were selected from among orthodontic patients attending consultant clinics and postgraduate orthodontic teaching clinics at Newcastle Dental Hospital. The hypodontia subjects study group was further divided into three subgroups that represented varying degrees of hypodontia—mild, moderate, and severe. All groups were balanced with regard to sex, age, and group size. Syndromes, history of orthodontic treatment and attrition or dental wear were exclusion criteria for selection of hypodontia subjects. Ethical approval for the study was granted by the County Durham and Tees Valley 1 Research Ethics Committee.

The following definitions and criteria were used to select subjects for the study groups:

Mild hypodontia (Group M): Cases with hypodontia of one or two teeth, excluding the third molars (20 males and 20 females).
Moderate hypodontia (Group D): Cases with hypodontia of three to five teeth, excluding the third molars (20 males and 20 females).
Severe hypodontia (Group S): Cases with hypodontia of six or more teeth, excluding the third molars (20 males and 20 females).
Control group (Group C): Cases with a full complement of the permanent dentition (20 males and 20 females).

### Data acquisition

Only pre-treatment study models of the maxillary and mandibular arches were used to examine tooth size and shape. Study models had already been made for all participants as part of a routine orthodontic assessment.

### Landmark definition and identification

**Figure 1:**
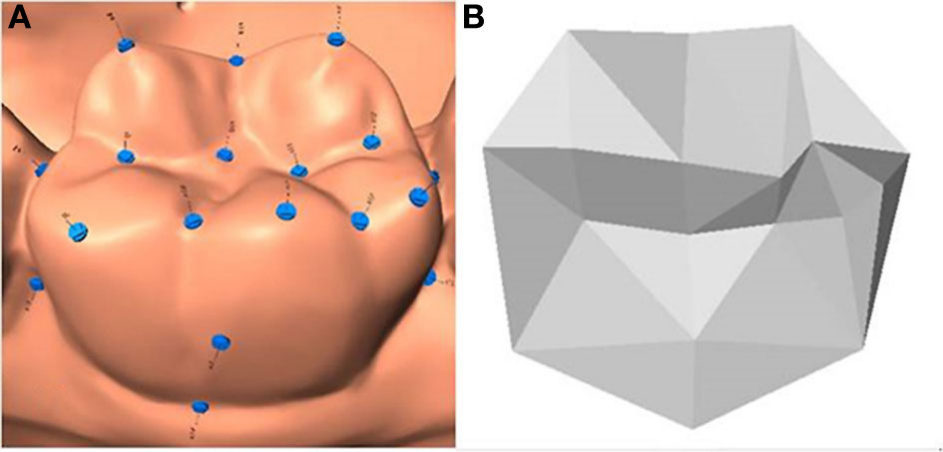
**Digitized Anatomical landmarks for lower left first permanent molar using freeware program called Landmark.exe**. **(A)** Scanned image. **(B)** Texture map.

Anatomical landmarks provide the core information on morphology in GMM. They have to be accurately defined, precise and relate to the same anatomical features in every specimen (Robinson et al., 2002; Oxnard and O'Higgins, 2009). Landmarks were defined according to tooth type or according to morphological class based on the descriptions of Robinson et al. (2002). Most of the landmarks were of Type I (anatomical evidence) and some were of Type II (geometric evidence) (Robinson et al., 2002; Zelditch et al., 2004). The different classes used in this project are detailed in Table 1. All study models were scanned using a 3D dental scanner (R640). Eighteen landmarks were defined and located on the lower left first permanent molar (Table 1). Each landmark was digitized using freeware program called Landmark.exe (Figure 1). Landmark 3D coordinates extracted from the data using Landmark.exe were saved as data text files for the subsequent analysis. The entire procedure (i.e., scanning process and landmarks digitization) on the lower left first molars from 20 sets of study models was repeated twice on two separate occasions with an interval of at least 2 weeks to estimate the measurement error using a hierarchical analysis of variance; the test called Procrustes ANOVA (Klingenberg et al., 2002). Shape matching considers the total configuration of landmarks, rather than individual landmarks. For example, the lower first molar has 18 landmarks with three coordinates on each landmark, and a total of 36 variables. This means we have only one shape unit rather than 36 variables. This approach is different from those adopted in traditional morphometrics, where each variable is treated separately. It may seem unclear to treat the entire shape as a single unit, but according to Zelditch et al. (2004), the rigor and power of these methods and their ability to visualize shape variation graphically overcomes this problem.

**Table 1:** **Defined anatomical landmarks for lower left first permanent molar**.

**Landmarks**	**Definition**
(1) and (2)	Mesial and distal (MD) contact points
(3)	Buccal endpoint of buccal lingual axis
(4) and (5)	Mesial and distal lingual cusp-tips
(6) and (7)	Mesial and distal buccal cusp-tips
(8)	Distobuccal cusp tip
(9–12)	Outer mesial, inner mesial, central and distal pits
(13) and (15)	Ends of mesial and distal papillae from the buccal side
(14)	Halfway between (13) and (15) along the gingival margin from the buccal side
(16)	Occlusal limit of buccal groove
(17)	Occlusal limit of distobuccal groove
(18)	Occlusal limit of lingual groove

### Data analysis

**Figure 2:**
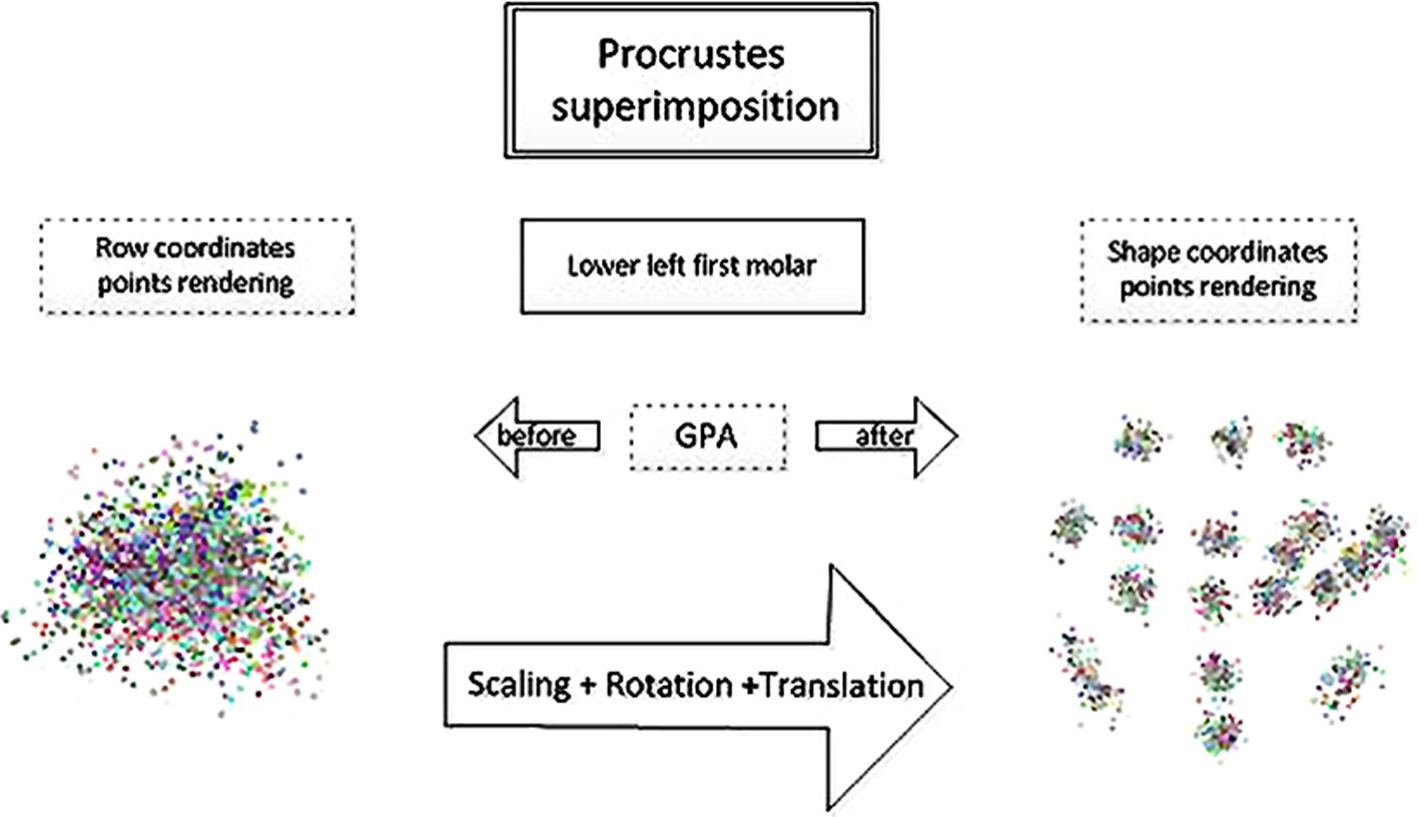
**Procrustes superimposition for the lower left first molar**.

Three-dimensional landmark coordinates (x, y, z) extracted from the data using Landmark.exe were submitted to the freeware shape analysis program MorphoJ (Klingenberg, 2010) to perform a Generalized Procrustes Analysis (GPA) to compute a new set of coordinates, called Procrustes shape coordinates (Figure 2). The aim of GMM is to extract relevant information and discard information that is not of interest. In this research, centroid size (CS)—the square root of the sum of the squared distances from each landmark to the centroid of the configuration (Bookstein, 1997; Dryden and Mardia, 1998)—was used to represent crown size. The GPA scales to unit CS, translates configurations to a common origin and rotates specimens to minimize differences in their relative positions using a least squares algorithm. The Procrustes shape coordinates capture the intrinsically multivariate nature of shape, and these data are analyzed using multivariate statistics in order to describe and compare shapes. The number of coordinates is more than the actual number of shape variables after the superimposition which explains the redundancy in the data. In three dimensional analyses as in this study, seven degrees of freedom are lost: one for scale, three for translational axes and three degrees for rotational planes (Zelditch et al., 2004; Viscosi and Cardini, 2011).

#### Variation in groups and between sexes

Differences in size in the study groups by sex was tested by analysis of variance (Two-Way ANOVA). For shape, differences within and between sex and group variability were tested using a multivariate analysis of variance (MANOVA). First, the interaction effect between group and sexes was tested and if no interaction effect was found the test was repeated to determine the main effects of sex and group. In addition, discriminant analysis (DA) was used to construct a model that would enable us to discriminate most accurately with respect to a least squares criterion among control and hypodontia groups. The main aim was therefore to classify individuals in groups according to the type of the group. In DA, group predictions were based on a leave-one-out cross-validation.

#### Mean shape similarity relationships

The main directions of shape variation were visualized using diagrams. These included wireframes (a set of lines connecting pairs of landmarks), surface rendering and thin-plate spline (TPS) deformation grids. The grids were computed using an interpolation technique and, in three dimensions, they were reflected onto planes through the volume defined by the landmark configuration. By warping grids, differences between a reference or the starting shape and a target shape can be better visualized. Vectors of coefficients are produced which can be used to predict how grid lines may change because of the warping. Generally, in this study, the starting shape was the control mean shape and the target shape was one of the hypodontia groups mean shapes. The shapes for each group were computed, and were then imported into Morphologika2 v2.5 (O'Higgins and Jones, 1998) in order to carry out visualization of mean shapes.

## Results

### Measurement of error

The digitization effect for CS as well as the scanning effect was negligible, with a minimum variance explained compared to the total variance. The total error variance for CS was 1.85%.

The analysis of variance, using Procrustes sums of squares as a measure of overall variation in shape, showed that individual variation was significant in relation to both scanning and digitization effects (Table 2). This led to the conclusion that the total measurement effect (17.2%) was negligible, with a small variance explained compared to the individual variation.

**Table 2:** **Total error of shape for lower left first molar**.

**Effect**	**% Explained**	***SS***	***MS***	***df***	***F***	***P***
Individual	82.79	0.90107054	0.00080381	1121	14	<0.0001
Scanner error	6.23	0.06775842	5.74224E-05	1180	1.13	0.006
Digitization error	10.98	0.11954161	5.06532E-05	2360		

### Size analysis

**Figure 3:**
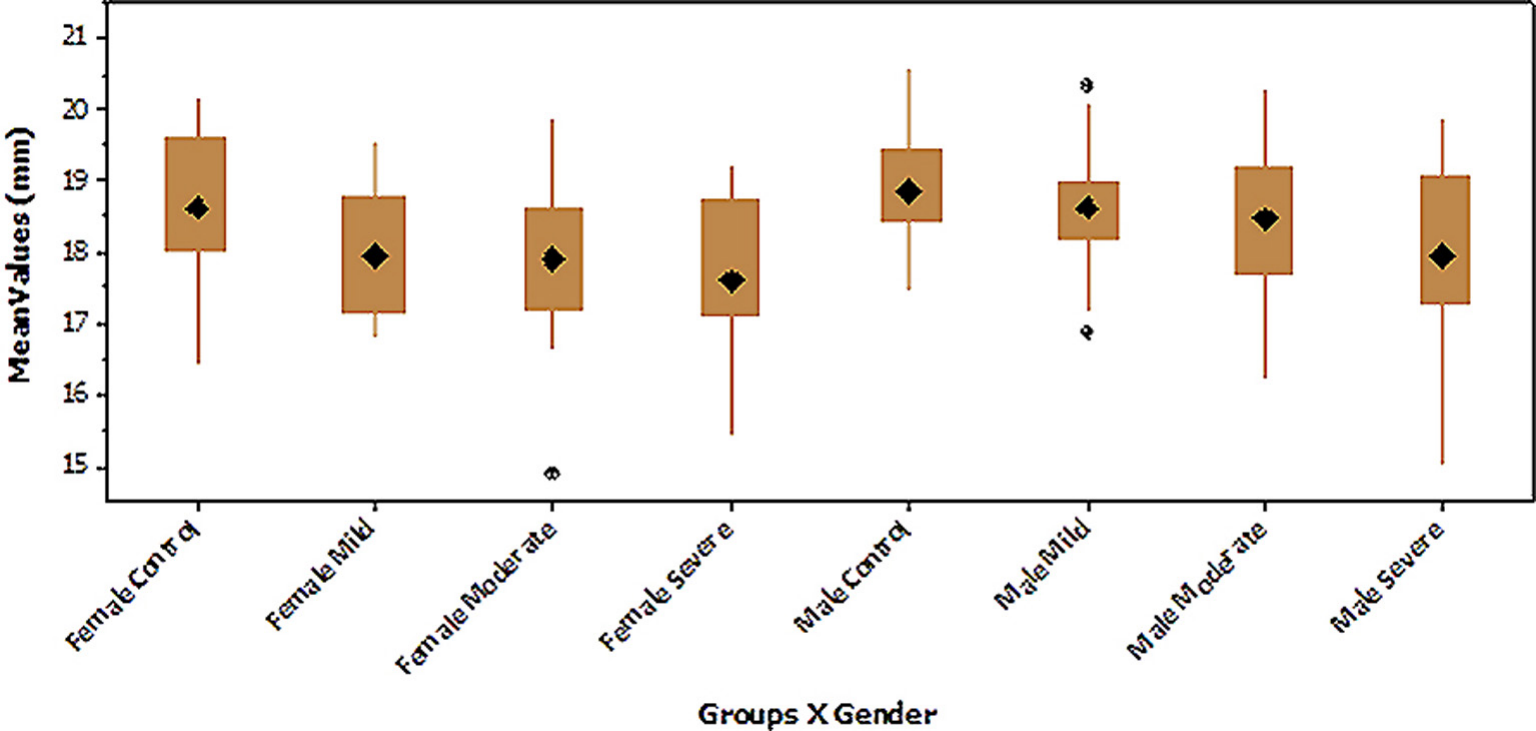
**Boxplot of tooth size of the lower first molar in the hypodontia and control groups with males and females analyzed separately**.

Descriptive statistics were computed for size with the groups split according to sex (i.e., the two sexes were treated separately) (Table 4). In the female subjects, the mean size was found to decrease from the control group through the mild, moderate, and severe hypodontia groups (Figure 3). The same pattern could be seen for the male subjects. The average size for male subjects was greater than that of females across all the four groups. Although there were two outliers within the male mild hypodontia group, the smaller inter-quarter range for this group indicated that it was less varied than the others. The female moderate and male severe hypodontia groups had the largest size range, indicating that the data were more varied. The coefficient of variation ranged between 5 and 7% for the female groups. The corresponding figures for male groups were between 4 and 9% (Table 3).

**Table 3:** **Descriptive statistics of tooth size of the lower first molar in the hypodontia and control groups with males and females analyzed separately**.

**Sex**	**Groups**	**Mean (mm)**	***SD***	***CV* (%)**	***N***
Female	Control	18.58	1.04	5.60	20
	Mild	17.88	0.9	5.03	20
	Moderate	17.84	1.11	6.22	20
	Severe	17.51	1.17	6.68	19
Male	Control	18.81	0.79	4.20	20
	Mild	18.57	0.84	4.52	20
	Moderate	18.39	1.09	5.93	19
	Severe	17.82	1.53	8.59	15

*SD, standard deviation; CV, coefficient of variation; N, number of first molars in each subgroup*.

#### Size variation in groups and between sexes

**Figure 4:**
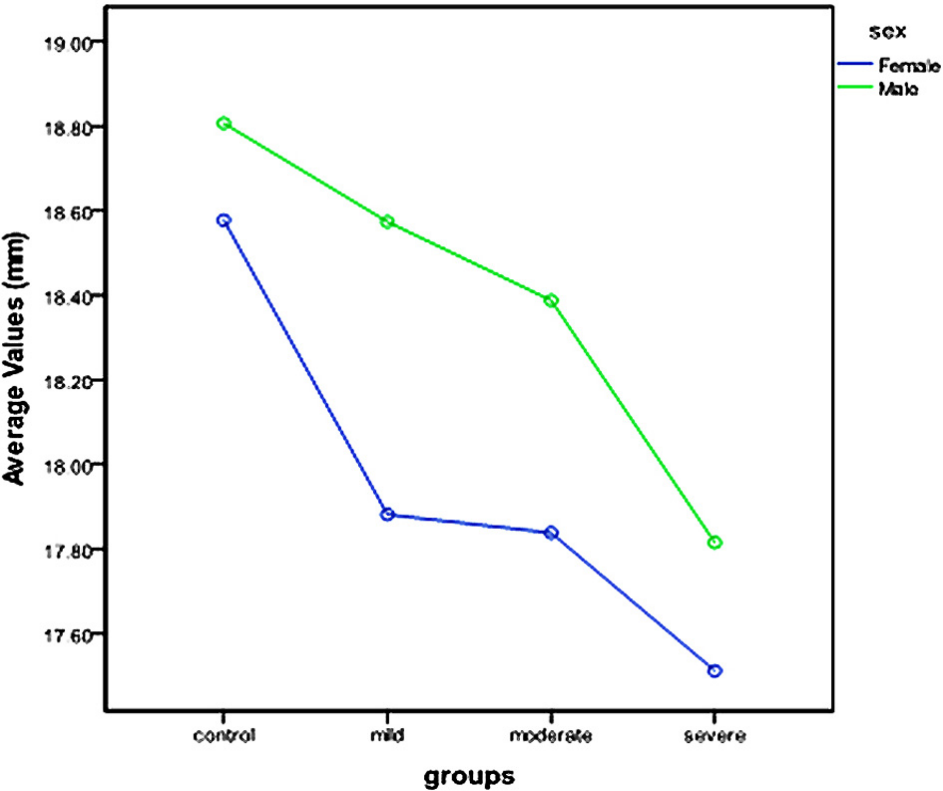
**Profile plot of tooth size of the lower first molar in the hypodontia and control groups with males and females analyzed separately**.

The results of the ANOVA (groups by sex) for size indicated that there was no significant interaction of group by sex (*p* < 0.05, Table 4). This is further suggested by the profile plots for mean size, which show almost parallel lines (Figure 4). The main effects of both group and sex were found to be statistically significant (*p* > 0.05, Table 4).

**Table 4:** **ANOVA of tooth size of the lower first molar of the hypodontia and control groups in both sexs**.

**Source**	***SS***	***df***	***MS***	***F***	**Sig**.
Groups	19.666	3	6.555	5.816	*0.001*
Sex	7.463	1	7.463	6.621	*0.011*
Groups ^*^ sex	1.353	3	0.451	0.4	0.753

*SS, the sum of the squares; df, degrees of freedom; MS, the mean square; F, the F ratio; Sig, the p-value*.

As there was no interaction effect of group by sex, the interaction term was removed and the ANOVA test was repeated. Again, the main effect of group and sex was found to be statistically significant (*p* < 0.05, Table 5).

**Table 5:** **ANOVA of groups by sex without the interaction term**.

**Source**	***SS***	***df***	***MS***	***F***	**Sig**.
Groups	19.469	3	6.49	5.829	*0.001*
Sex	7.682	1	7.682	6.899	*0.010*

Because of this significant difference found between the sexes, size was then examined within groups by sex. The results are presented in the following section. Non-parametric permutation tests were carried out pairwise with split-sex samples. Among the female participants, only the severe hypodontia group was found to differ significantly from the control group after a sequential Bonferroni correction, with 19.67% of the variance explained by group membership (Table 6). The same finding was seen in the males, wherein severe hypodontia group showed significant differences in size as well, with 15.86% of the variance explained by group membership (Table 7).

**Table 6:** **Pairwise comparison of female group tooth size averages**.

	**Control**	**Mild**	**Moderate**	**Severe**
Control	−	11.52%	11.09%	19.67%
Mild	0.0358	−	3.21%	3.21%
Moderate	0.0355	0.2664	−	2.11%
Severe	*0.0050*	0.2764	0.3757	−

*In this and all other tables for pairwise tests, p-values, estimated using 10,000 random permutations, are shown below the main diagonal and percentage of variance explained by group membership; p-values significant after a sequential Bonferroni correction for multiple comparisons are shown in italics*.

**Table 7:** **Pairwise comparison of male group tooth size averages**.

	**Control**	**Mild**	**Moderate**	**Severe**
Control	−	2.11%	4.87%	15.86%
Mild	0.3688	−	0.96%	9.68%
Moderate	0.1752	0.5562	−	4.83%
Severe	*0.0081*	0.0693	0.2137	−

### Shape analysis

A principal component analysis (PCA), which identifies the maximum variation within the sample, was performed in order to reduce the dimensionality in the analysis.

**Figure 5:**
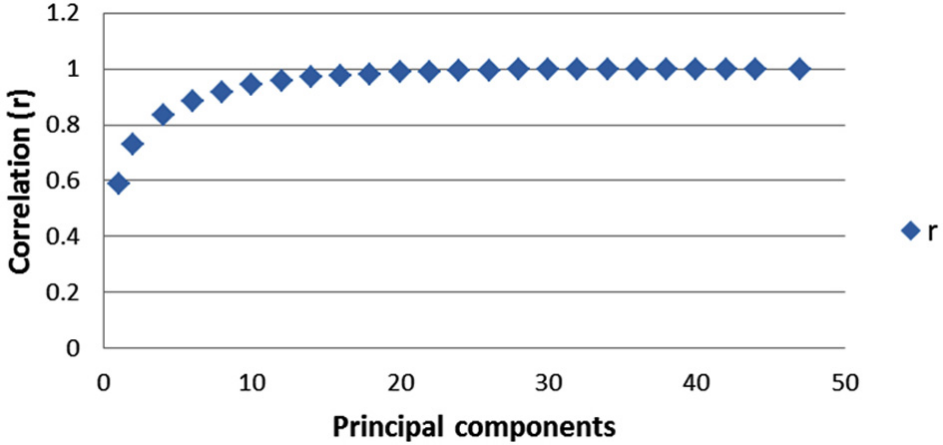
**Plot of the values of the correlation coefficient (r) between Procrustes shape distances and the Euclidian distances as a function of the number of PCs included, from 1 up to 47 PCs**. 20 PCs, explaining 84% of the total variance, with *r* = 0.988, retained in the analysis of shape.

The first 20 principal components (PCs) explained approximately 84% of total shape variance, and the correlation of Euclidean distances based on these 20 PCs and Procrustes shape distances in the full shape space was larger than 0.98. The inverted scree-plot based on correlations between shape distances showed that the correspondence between the shape space distances of reduced dimensionality and the full shape space did not increase appreciably after including 20 PCs (Figure 5). Thus, the first 20 PCs were used for parametric tests of differences between groups.

The MANOVA sex by groups for shape (first 20 PCs) showed that all factors including their interaction were highly significant *p* < 0.05 (Table 8).

**Table 8:** **MANOVA of groups by sex**.

**Effect**	**Wilks' Lambda**	***F***	**Hypothesis *df***	**Error *df***	**Sig**.	**Eta Squared**
Groups	0.143	5.762	60	376.75	0.001	0.477
Sex	0.698	2.723	20	126	0.001	0.302
Groups* sex	0.491	1.69	60	376.75	*0.002*	0.211

#### Group differences

Split-sex samples were used in all the analyses, because in the MANOVA sexual dimorphism was found to be significant and the pattern of group shape differences was different between the sexes (significant interaction). Pairwise tests for differences between groups using Procrustes distance after a sequential Bonferroni correction virtually always showed significant differences. On an average, the differences explained 7–10% of shape variance.

Among the females, significant differences were found in 100% of the pairwise comparisons across groups after a sequential Bonferroni correction, and on average 10% of the variance was explained by group membership (Table 9). The largest differences were found between the mild and moderate/severe hypodontia groups followed by those between the control and moderate/severe hypodontia groups which were fairly small. All these differences were statistically significant.

**Table 9:** **Pairwise tests for mean shape differences between female groups**.

	**Control**	**Mild**	**Moderate**	**Severe**
Control	−	11.20%	6.61%	7.65%
Mild	*<0.0001*	−	18.27%	18.27%
Moderate	*<0.0001*	*<0.0001*	−	6.65%
Severe	*<0.0001*	*<0.0001*	*0.0002*	−

**Figure 6:**
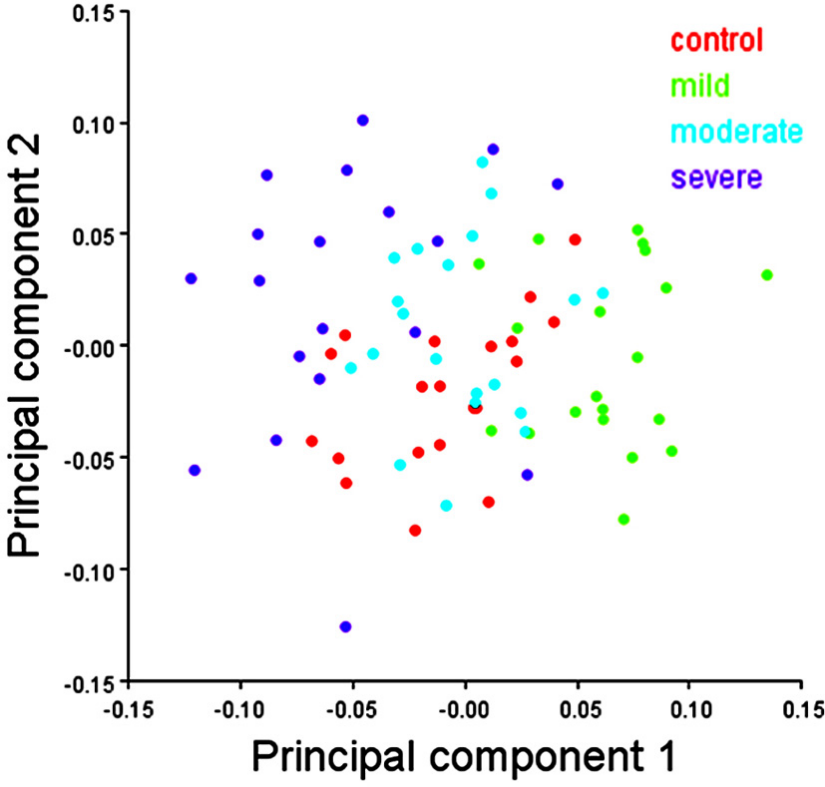
**Scatter plots of the first two principal components (PCs) of shape variables in females**. The first and second PCs represents 16.46 and 11.74% of total shape variance, respectively.

In females, the scatter plot of the first two PCs of shape showed a clear separation between the mild and severe hypodontia groups and a large overlap between the control and moderate hypodontia groups for PC1 (Figure 6). For PC2 and further PCs (not shown here), the groups largely overlapped.

Among the males, the results of all pairwise comparisons except one were found to be significant after a sequential Bonferroni correction, and on average 7.41% of the variance was explained by group membership. The only non-significant pairwise comparison was that between the mild and moderate hypodontia groups (Table 10). The largest differences were found between the control and severe hypodontia and also between the mild and severe hypodontia groups.

**Table 10:** **Pairwise tests for mean shape differences between male groups**.

	**Control**	**Mild**	**Moderate**	**Severe**
Control	−	6.03%	5.82%	10.86%
Mild	*0.0022*	−	4.11%	10.68%
Moderate	*0.0006*	0.0370	−	6.95%
Severe	*<0.0001*	*<0.0001*	*0.0036*	−

**Figure 7:**
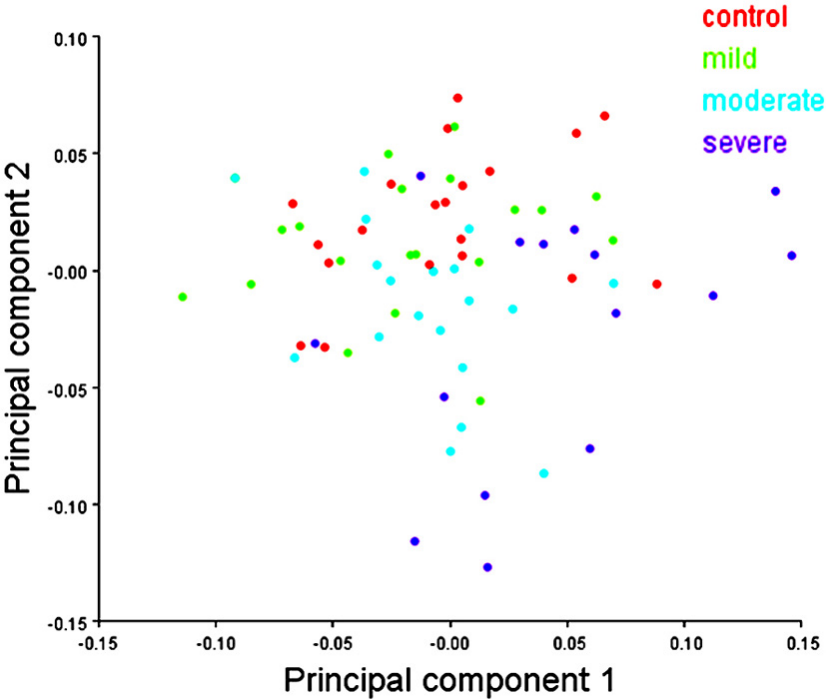
**Scatter plots of the first two principal components (PCs) of shape variables in males**. The first and second PCs represents 16.99 and 10.98% of total shape variance, respectively.

In males, the scatter plot of the first two PCs of shape showed a slight separation of the severe hypodontia group from the remaining groups and a large overlap among the control, mild, and moderate hypodontia groups (Figure 7) for PC1. For PC2 and further PCs (not shown here), the groups largely overlapped.

#### Mean shape similarity relationships

**Figure 8:**
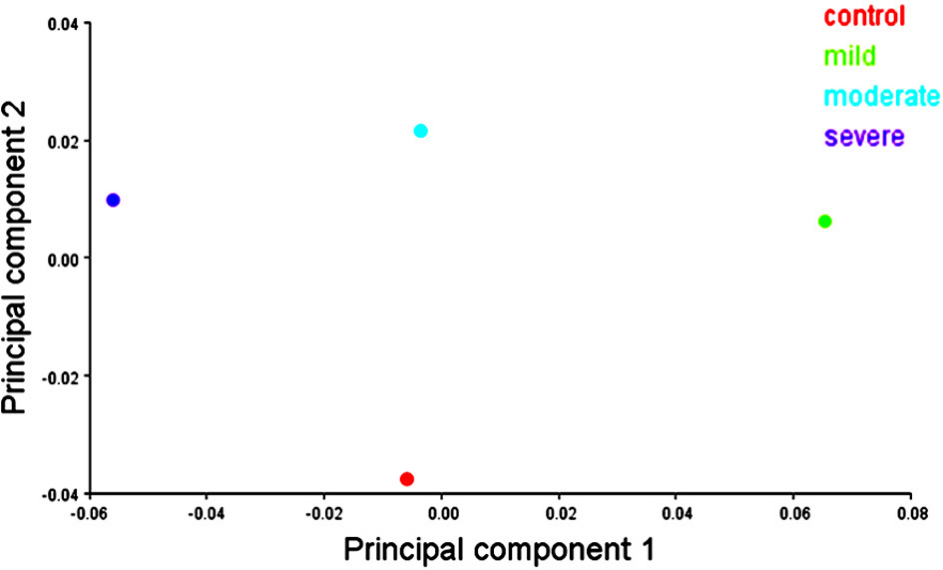
**Scatter plots of the first 2 principal components (PCs) of mean shape variables in females**.

**Figure 9:**
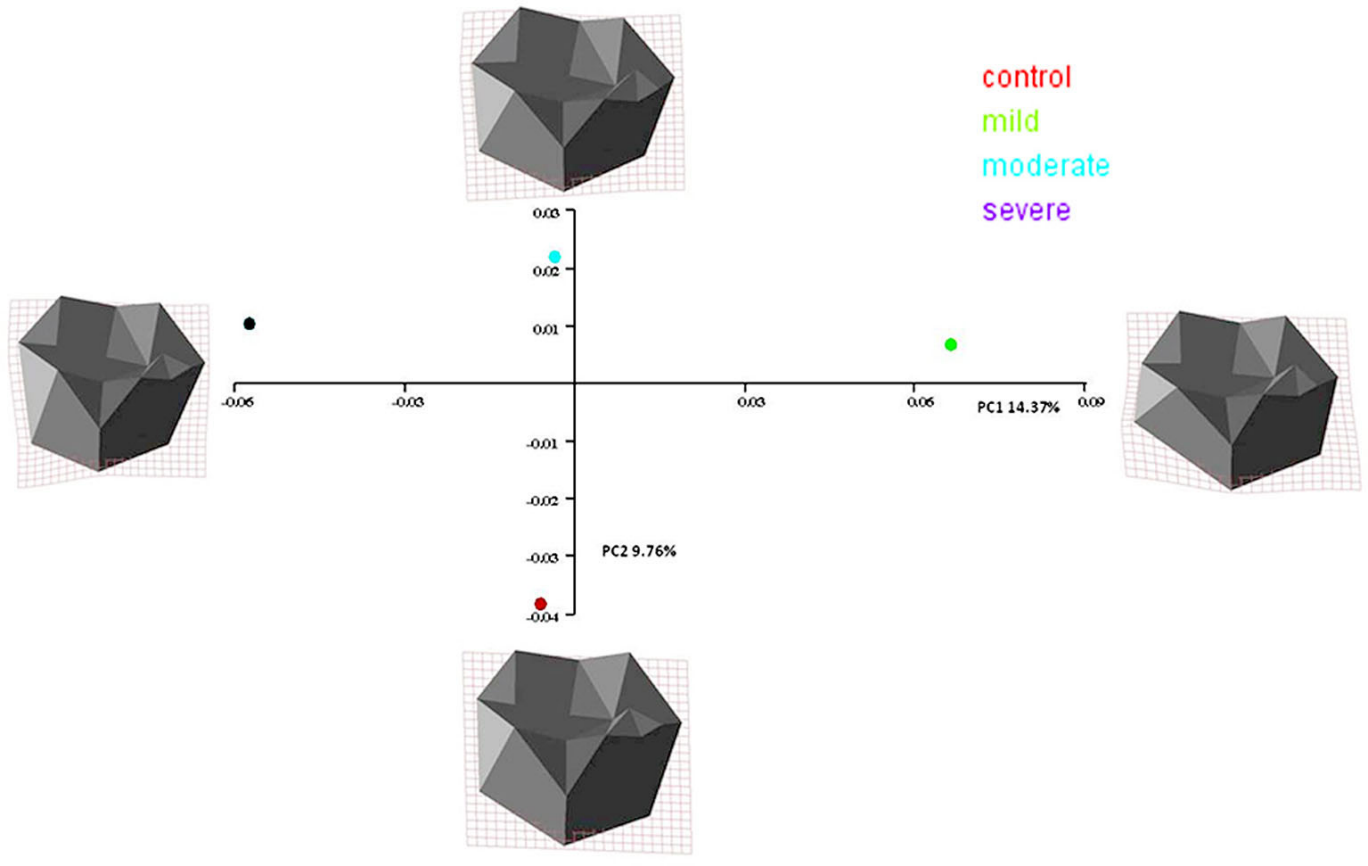
**Transformation grids for female groups mean shape using thin-plate spline derived from the difference between the reference form (control mean shape) and the various target forms (hypodontia groups' mean shape)**.

**Figure 10:**
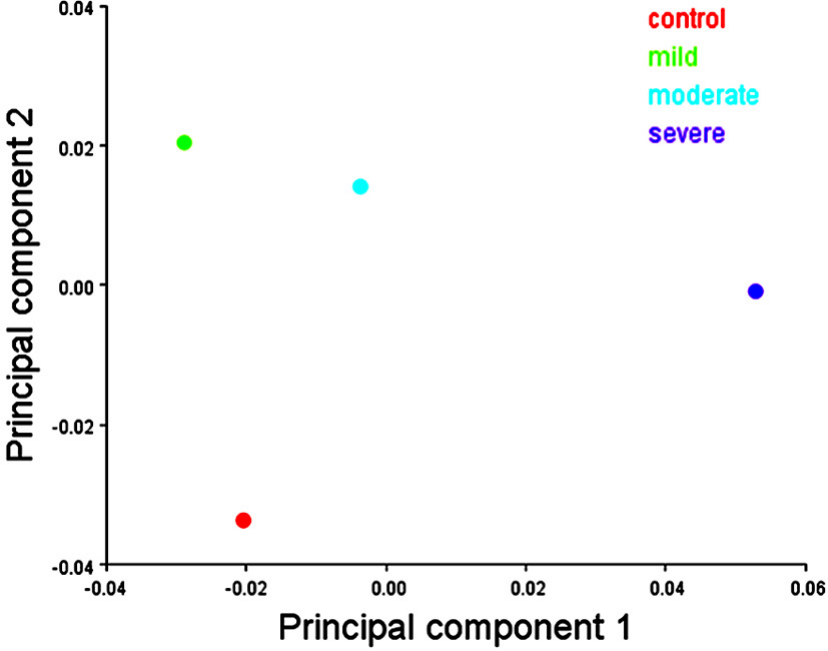
**Scatter plots of the first 2 principal components (PCs) of mean shape variables in males**.

**Figure 11:**
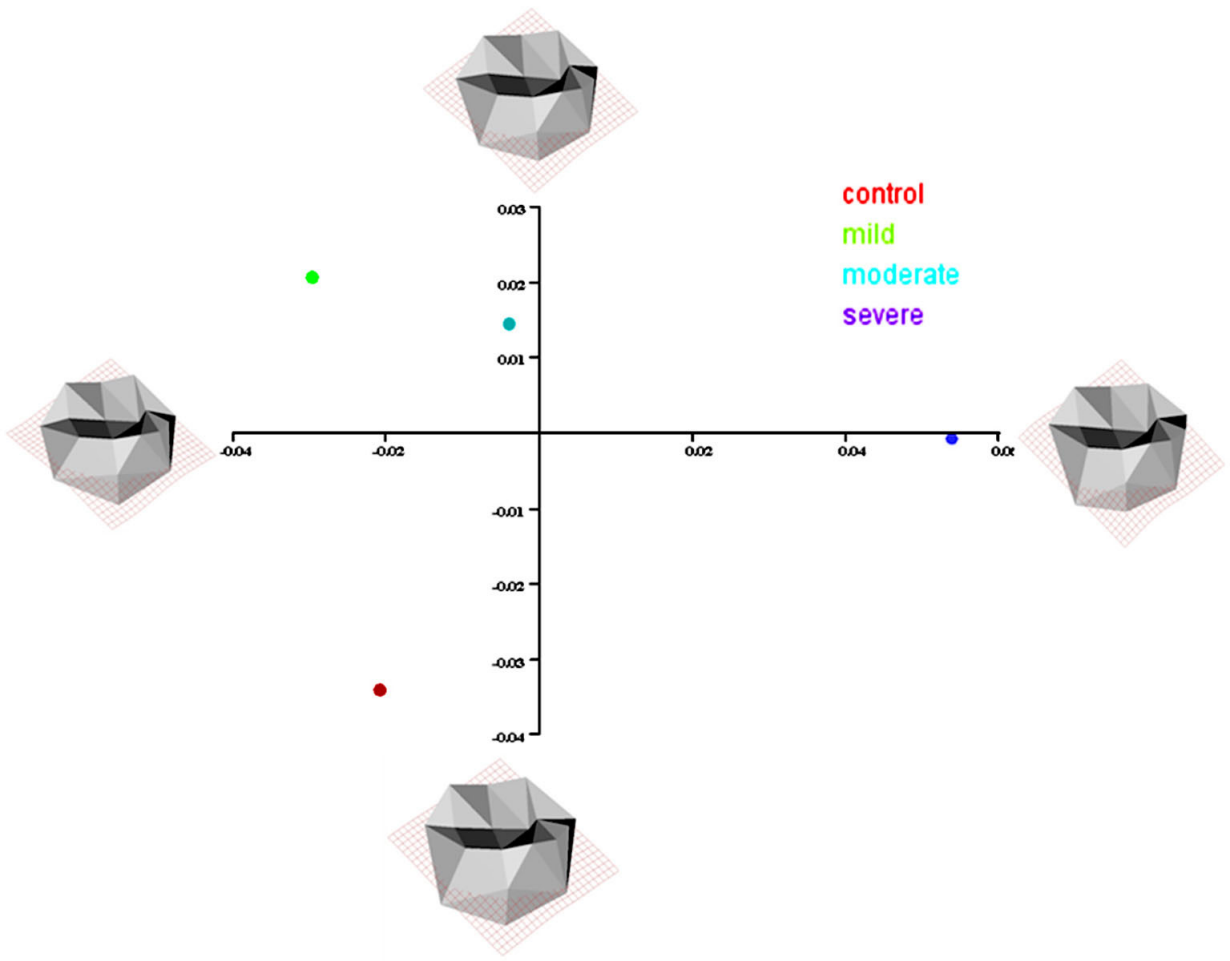
**Transformation grids for male groups mean shape using thin-plate spline derived from the difference between the reference form (control mean shape) and the various target forms (hypodontia groups' mean shape)**.

Similarity relationships among groups per sex were summarized using a PCA on the matrix of mean shape variables. A mean shape is computed by taking the sample average of shape coordinates from the full set of shape variables. In females, PC1 differentiated the hypodontia groups progressively according to the increasing degree of severity, while PC2 separated the control group from the hypodontia groups (Figure 8). The hypodontia groups showed a progressive shortening at the gingival margin. In addition, the gingival margin became flatter, with longer and more divergent proximal surfaces particularly the mesial one as one moved from the mild toward the severe hypodontia groups. However, shape variation on the vertical axis can be summarized by comparing the control against the hypodontia groups. The hypodontia groups were found to have a flatter occlusal plane with slightly smaller cusps particularly the disto-lingual cusp when compared to the control subjects (Figure 9). The same analysis was performed for the males (Figures 10, 11); the results suggested the same group pattern and showed similar shape differences among groups.

#### Allometry

The effect of size on shape (allometry) was tested by regressing shape onto CS within each of the eight groups (Table 11). The effect was found to be significant only in the male moderate hypodontia group with a *p*-value of 0.0255 and about 9% of shape variance explained by size. With a sequential Bonferroni correction, however, the effect for this group was not found to be significant. The allometric variance across all the eight groups ranged from a minimum of nearly 5% to a maximum of nearly 9%. Although the evidence for allometric variation was very weak, a MANCOVA model was used to test for differences after holding just a small effect of size on shape constant.

**Table 11:** **Group regression of tooth shape onto size**.

**Sex**	**Groups**	**%**	***P*-value**
Female	Control	7.05	0.1171
	Mild	6.20	0.2325
	Moderate	7.48	0.0649
	Severe	5.37	0.4877
Male	Control	6.31	0.2102
	Mild	4.72	0.5855
	Moderate	8.81	*0.0255*
	Severe	8.51	0.2569

*P-value, estimated using 10,000 random permutations*.

A MANCOVA conducted using the first 20 PCs of shape showed that there was no interaction effect of size on groups for either female or male subjects (Table 12). Thus, the effect of size on shape was similar across groups. The interaction term was therefore removed and the analysis repeated using only groups (without the interaction term) and the size covariate.

**Table 12:** **MANCOVA of groups across sex onto size with interaction**.

**Sex**	**Effect**	**Wilks' Lambda**	***F***	**Hypothesis *df***	**Error *df***	**Sig**.	**Eta Squared**
Female	Groups	0.407	0.915	60	155.974	0.647	0.259
	CS	0.638	1.472	20	52	0.133	0.362
	Groups^*^ CS	0.417	0.885	60	155.974	*0.701*	0.253
Male	Groups	0.344	1.01	60	141.057	0.471	0.299
	CS	0.466	2.689	20	47	0.003	0.534
	Groups^*^ CS	0.347	1.001	60	141.057	*0.487*	0.297

*CS, Centroid size*.

The results of the MANCOVA analysis without the interaction effect indicated that the main effect of group was highly significant for both females and males (Table 13). Allometric variation was small (similar in both sexes), but differences between groups for both male and female subjects were significant. This indicates that allometric patterns are similar but laterally transposed (i.e., with parallel lines shifted up or down relative to one another). To examine group variation further, when the effect of size on shape was held constant, shapes were “size-corrected” and the test of group differences re-run.

**Table 13:** **MANCOVA of groups across sex onto size without interaction**.

**Sex**	**Effect**	**Wilks' Lambda**	***F***	**Hypothesis *df***	**Error *df***	**Sig**.	**Eta Squared**
Female	Groups	0.065	4.106	60	164.924	*0.001*	0.597
	CS	0.628	1.632	20	55	0.078	0.372
Male	Groups	0.077	3.406	60	150.007	*0.001*	0.575
	CS	0.436	3.229	20	50	0.001	0.564

*CS, Centroid size*.

#### “Size-corrected” pairwise comparisons

The results of the pairwise tests for group differences after controlling for allometry were exactly the same in both sexes as those for full shapes. This is consistent with the observation of a small and probably negligible effect of size on shape. A significant difference was found between groups across all possible pairwise comparisons after a sequential Bonferroni correction. The magnitude of group differences ranged between 7 and 18% among all groups (Table 14).

**Table 14:** **Pairwise tests for mean shape between female groups after size correction**.

	**Control**	**Mild**	**Moderate**	**Severe**
Control	−	12.20%	6.61%	7.65%
Mild	*<0.0001*	−	10.57%	18.27%
Moderate	*0.0001*	*<0.0001*	−	6.65%
Severe	*0.0018*	*<0.0001*	*0.0012*	−

A significant difference was also found between male groups across all possible pairwise comparisons, with the exception of the pairwise test between the mild and the moderate hypodontia groups. The difference between these two groups was found to be non-significant after a sequential Bonferroni correction. The magnitude of differences varied between 5 and 11% (Table 15).

**Table 15:** **Pairwise tests for mean shape between males groups after size correction**.

	**Control**	**Mild**	**Moderate**	**Severe**
Control	−	6.03%	5.82%	10.86%
Mild	*0.0025*	−	4.11%	10.68%
Moderate	*0.0016*	0.045	−	6.95%
Severe	*<0.0001*	*<0.0001*	*0.0041*	−

#### “Size-corrected” discriminant analyses

The results of the discriminant analyses were highly significant: females Wilks' λ = 0.069, *F*_(60, 168)_ = 4.053 with a *p*-value of 0.0001; males Wilks' λ = 0.082, *F*_(60, 153)_ = 3.342 with a *p*-value of 0.0001. Overall, at least 76% of teeth were correctly classified in a priori specific sub-groups with the percentage for females being slightly higher (86%) than that for males (84%). When the results were cross-validated, however, the percentages of correctly classified specimens dropped to slightly over 64 and 54% in female and male groups, respectively. The results of the “size-corrected” shape analyses were virtually identical to those for the actual shape without size correction, which would be expected if the effect of size on shape were negligible.

## Discussion

Three main variation factors have been considered in this study: size, shape, and allometric variation. These factors were tested in comparisons between control subjects and hypodontia groups and between hypodontia groups themselves. Separate statistical models were built for each factor.

### Variation of tooth size in hypodontia patients

Statistically, it was found that in the hypodontia subjects the size of the lower first permanent molar was smaller than in the control group. The decrease in size was directly proportional to the degree of severity of hypodontia with the mean size values decreasing progressively from the control subjects through mild, moderate and then severe hypodontia. This is consistent with the results of previous studies that have employed traditional measurement techniques (Garn and Lewis, 1970; Baum and Cohen, 1971; Rune and Sarnas, 1974; Brook, 1984; Ooshima et al., 1996; Schalk-van der Weide and Bosman, 1996; Brook et al., 2002, 2009a; McKeown et al., 2002). Other researchers have reported that the degree of size reduction was associated with the degree of the severity of the hypodontia (Rantanen, 1956; Alvesalo and Portin, 1969; Garn and Lewis, 1970; Lavelle, 1970; Rune and Sarnas, 1974; Brook, 1984; Brook et al., 2009b; Yaqoob et al., 2011; Mirabella et al., 2012). In fact, this correlation between the findings obtained using this novel 3D GMM methodology and those obtained using the old traditional morphometrics validate the method but because 3D GMM includes shape as well, its use will help to fill the gap in our understanding of the etiological factors that lie behind the dental developmental process. Further research may help to establish a proper link between the early molecular events and the variation within the human dentition. The limited knowledge of shape that has been obtained by the metric and non-metric dental variables can be overcome by the use of GMM in conjunction with multivariate shape statistics. This knowledge will indeed greatly improve clinical practice in both diagnosis and treatment planning.

### Variation of tooth shape in hypodontia patients

It was found that the shape of the lower first molar differed from the control group in hypdontia patients. Furthermore, the more severe the hypodontia, the higher the degree of tooth shape differences. The shape of the molar in the hypodontia subgroups showed a progressive shortening of the clinical crown at the gingival margin. In addition, the gingival margin became flatter with a less bulbous labial surface as the shape warped from the control toward the hypodontia subjects. Furthermore, the buccal cusp tips of hypodontia subjects were less prominent than those of the control subjects for the lower first permanent molar. The proximal surfaces were less tapered toward the occlusal surface in hypodontia when compared to control subjects.

According to Kondo and Townsend (2006), shape variation in teeth is mainly due to genetic and environmental factors and these changes are expressed during crown development, congruent with the findings in Brook's review (2009). The stage of tooth morphogenesis within the development process controls the presence or absence and the size and shape of the individual tooth (Brook, 2009). Larger upper first molars tend to display Carabelli cusps, while smaller molars tend to have no or less developed Carabelli cusps (Kondo and Townsend, 2006). This is consistent with our results indicating that hypodontia patients, with their generally smaller lower first molar, tend to have flatter occlusal surfaces with less prominent cusps. Other researchers who have measured crown height (Miyabara, 1915; Bolton, 1958; Lavelle, 1968; Volchansky et al., 1981) or the crown shape index (Garn et al., 1967; Lavelle, 1968, 1970) found shorter crowns and smaller crown indices among hypodontia subjects. However, the nature of the measurements they used inevitably limited the amount of information they were able to capture concerning the morphology of the teeth. Robinson et al. (2001) applied Procrustes methods to images using the image-analysis system developed by Brook et al. (1998) to explore differences in the buccal surface of the upper central incisors. The results of their study indicated that the teeth of hypodontia patients were different in shape at the incisal corners, such that the incisors were more tapered toward the incisal edge than those of control subjects. Again their investigation was limited to only one surface, which was based on a 2D imaging system. The 3D GM method of analyzing and describing tooth shape variation used in the current study produces far more descriptive results than any of the methods employed in previous research in this area and thus enables the clinicians to see visually the degree of tooth shape variation between hypodontia groups and control subjects.

### Allometric variation of tooth size on shape

In the present study the differences found in shape of the lower left first molar between the hypodontia and control groups were not simply allometric in nature, because even after controlling for the effect of allometry in a MANCOVA, group differences were significant: for example, among the male moderate hypodontia subjects, the lower left first molar showed allometric variation; however, the “size-corrected” shape analyses were virtually identical to those for the actual shape without size correction, which would be expected if the effect of size on shape were negligible. This means that shape variation across groups is not simply a “side-effect” of size differences, which were large for most teeth. In conclusion, the allometric effect was found to be very small, which means that it cannot be considered a factor in causing the variations in crown morphology.

### Tooth size differences of the lower left first molar between sexes

The results of the present study revealed that the explained variance within the male severe hypodontia group was higher than that in female severe hypodontia groups when compared to their corresponding control subjects. Similarly, a recent study conducted by Brook et al. (2009a) found that, on average, the percentage reductions in the MD and BL dimensions were higher in male rather than female hypodontia patients, which indicates that the degree of tooth size variation was higher in the male subjects. This could be explained by Brook's model, 1984, 2009 that suggests that the teeth of male hypodontia subjects deviate further from the normal mean than those of females, i.e., the expression of tooth size reduction in hypodontia subjects is greater in males than in females.

### Tooth shape differences of the lower left first molar between sexes

Differences in tooth shape between the sexes were found in the lower first molar on both sides. This may be explained by the complexity of the shape of this tooth type and by the fact that congenital absence of the other teeth and/or microdontia may have a different etiology from that of the lower first molars. It may also be due to the fact that molars are only rarely missing in hypodontia as in this study only seven lower first molars were missing.

The percentage of shape differences varied between the sexes: greater differences were found between the female hypodontia groups and female control subjects than between the male hypodontia groups and male controls; however, the same pattern of shape variation was found when using the TPS visualization technique for the lower first molars. This may suggest that shape variation is expressed more in female than in male subjects.

### Clinical relevance of the study

In a recent review of the multivariate statistical approach to measuring teeth, Townsend et al. (2009) have pointed out that previous researchers have all used the traditional method of measuring crown diameters, and have suggested that new measurement techniques that provide more information about tooth form should be adopted. Landmark-based geometric morphometric methods (GMM) (Rohlf and Marcus, 1993; Adams et al., 2004; Zelditch et al., 2004; Baab and McNulty, 2009) capture the form of a structure, providing full information about the geometry of the tooth; this geometry is impossible to quantify using traditional methods. Size and shape of teeth have been described using traditional tools limited to selected dental variables or very simple indices. The collected information is limited and does not describe dental variation visually. With developments in the imaging field (e.g., 3D scanners) and the increased knowledge of multivariate shape statistics (i.e., GMM), it has been possible to overcome those limitations and describe dental variation clinically with a high degree of accuracy and precision. The knowledge acquired not only provides improved clinical discrimination but also leads to new lines of research to obtain a better understanding of the underlying developmental processes that occur during odontogenesis. In addition, helping to differentiate between different groups may also allow us to investigate the contributions of different etiological factors such as genetic, epigenetic and environmental factors to observed variation (Townsend et al., 2011).

The consequences of the congenital absence of teeth may be both physical and emotional, especially if teeth are missing in the anterior region (Hobkirk et al., 2011). The congenital absence of teeth requires extensive care by a multidisciplinary team. The multidisciplinary team works together to devise the best treatment plan and delivery of care for the management of patients. The role of the dental team is to maintain the remaining dentition, improve aesthetics, improve function, and promote psychological and emotional well-being and to encourage the acceptance of such patients by their families and peers. However, treatment is dependent on the pattern of tooth absence, the presence and severity of the microdontia and abnormal tooth shape, the amount of residual spacing, the presence of malocclusion and the attitude of the patient (Valle et al., 2011). The initial steps, including a diagnostic wax up with a good set of models and radiographs, will certainly lead to clear planning (McNamara et al., 2006).

Clinically, a good knowledge of the size and shape of each tooth enables the clinician to form the provisional and future definitive treatment plans. Furthermore, quantifying tooth shape provides valuable information for evaluating the final tooth position and morphology. The present findings have revealed a general trend for the lower left first molar to have flatter buccal surfaces than the teeth of control subjects. At the moment, when brackets are used for hypodontia patients the final tooth position is not optimal, since the built-in prescriptions are based on the tooth shape of control patients. Also, it would be useful for clinicians to have some knowledge of the consequences of dental anomalies, as many studies have demonstrated a correlation between congenitally missing teeth and delayed eruption, ectopic eruption, malposition, taurodontism, rotation of teeth, short teeth, and arch length form alteration (Baccetti, 1998).

## Conclusion

In conclusion, our study has demonstrated that a new, comprehensive 3D method based on landmark configuration could prove to be more useful and reliable than 2D methods in quantifying size and shape differences between patients with hypodontia and those with normal dentitions. This method allows not only size, but also shape differences to be quantified. Generally the more severe the hypodontia, the higher the degree of tooth shape differences. While males show significantly larger mean centroid sizes than females, the effect of size on shape is negligible. Hypodontia patients show a progressive shortening of the clinical crown of the lower first molar, with less prominent buccal cusp tips and more occlusally tapered proximal surfaces. The use of GMM may improve clinical outcomes as shape differences are captured in a way that 2D methods cannot provide.

### Conflict of interest statement

The authors declare that the research was conducted in the absence of any commercial or financial relationships that could be construed as a potential conflict of interest.
